# Regulating Strategies for Producing Carbohydrate Active Enzymes by Filamentous Fungal Cell Factories

**DOI:** 10.3389/fbioe.2020.00691

**Published:** 2020-07-08

**Authors:** Teng Zhang, Hu Liu, Bo Lv, Chun Li

**Affiliations:** ^1^Institute for Synthetic Biosystem/Department of Biochemical Engineering, School of Chemistry and Chemical Engineering, Beijing Institute of Technology, Beijing, China; ^2^Key Laboratory of Systems Bioengineering (Ministry of Education), Collaborative Innovation Center of Chemical Science and Engineering (Tianjin), School of Chemical Engineering and Technology, Tianjin University, Tianjin, China; ^3^Key Lab for Industrial Biocatalysis, Ministry of Education, Department of Chemical Engineering, Tsinghua University, Beijing, China

**Keywords:** filamentous fungal cell factory, protein expression, carbohydrate active enzymes, transcription factors, carbon catabolite repression, signal pathway, regulation strategies

## Abstract

Filamentous fungi are important eukaryotic organisms crucial in substrate degradation and carbon cycle on the earth and have been harnessed as cell factories for the production of proteins and other high value-added products in recent decades. As cell factories, filamentous fungi play a crucial role in industrial protein production as both native hosts and heterologous hosts. In this review, the regulation strategies of carbohydrate active enzyme expression at both transcription level and protein level are introduced, and the transcription regulations are highlighted with induction mechanism, signaling pathway, and promoter and transcription factor regulation. Afterward, the regulation strategies in protein level including suitable posttranslational modification, protein secretion enhancement, and protease reduction are also presented. Finally, the challenges and perspectives in this field are discussed. In this way, a comprehensive knowledge regarding carbohydrate active enzyme production regulation at both transcriptional and protein levels is provided with the particular goal of aiding in the practical application of filamentous fungi for industrial protein production.

## Introduction

In recent years, green biomanufacturing is getting increasing attention because of the serious energy crisis and environment pollution. Searching for green and environmentally friendly ways for industrial production has gradually become a new theme for human beings, such as converting hardly degradable biomass into fermentable sugars and ethanol, as well as replacing fossil energy with environmentally friendly clean energy. However, the transformation of biomass requires a huge number of industrial enzymes, especially carbohydrate active enzymes (CAZymes), such as cellulases and amylases. It is crucial to attain better cost performance or improved properties for these industrial enzymes. Microorganisms show irreplaceable advantages in bioeconomy and green biomanufacturing for their fast growth, short culture period, and low culture cost. Filamentous fungi, which widely exist on earth, play a crucial role in global carbon cycle for their excellent performance in degrading organic matters and converting plant biomass into cost-effective fermentable sugar. In addition, filamentous fungi can also produce a lot of secondary metabolites that could be used as antibiotics, such as penicillin, or some other drugs that are applied in tumor therapy. Moreover, they can produce many organic acids and other chemical materials.

Altogether, the application of fungal biotechnology enables the development of many industrial fields, such as enzyme and pharmaceutical production, biofuels and biochemistry, food, agriculture, pulp and paper, detergents, textiles, and crop protection. As a consequence, filamentous fungi are getting increasing attention for their major role in industrial production (Dunford, 2012; Deacon, 2013; Benocci et al., 2017; Fang and Qu, 2018; Jiang et al., 2018). As eukaryotic cell factory, filamentous fungi can presumably serve as ideal hosts with rapid growth rate on simple and inexpensive media.

There are several kinds of hosts for protein expression, including both prokaryotic and eukaryotic protein expression system (Altmann et al., 1999; Balbas and Lorence, 2004; Kantardjieff and Zhou, 2013; Schmoll and Dattenböck, 2016; Vega, 2016), and their comparison is listed in Table 1. As eukaryotic cell factory, filamentous fungi have numerous advantages that cannot be replaced by other organisms. For instance, filamentous fungi can grow rapidly on simple and cheap media and even fermented or unfermented agroindustrial wastes. They also have a strong survivability, which make them an ideal cell factory for producing drugs, antibiotics, industrial enzymes, and other substances. Most importantly, filamentous fungi have a strong ability in protein expression and perform various posttranslational processing correctly, including glycosylation, peptide chain shearing, and disulfide bond formation, which are similar to mammal cells (Bergquist et al., 2002). Besides, filamentous fungi have a powerful secretory pathway, including signal recognition particle signaling and efficient function of the endoplasmic reticulum in protein modification, as well as rapid clearance of misfolded proteins, fusion between vesicles and target membranes, and apical secretion of the proteins, which conferred them the ability to produce eukaryotic proteins correctly (Kavanagh, 2011; Karagiosis and Baker, 2012; Fang and Qu, 2018).

**TABLE 1:** Comparison of common expression hosts.

Organisms	Growth and culture condition	Genetic transformation	Posttranslational modification	Expression efficiency	Cost
**Prokaryote**					
*E. coli*	Fast and high efficiency, simple media requirement	Well-defined, simple, and high efficiency	No posttranslational	High without efficient secretion	Low cost
*Bacillus subtilis*	Fast, high efficiency, and safe	Convenient for gene modification	Almost none	High yield with secretory expression and produces no lipopolysaccharide	Low cost
**Eukaryote**					
*S. cerevisiae*	Fast and high efficiency, easy scale-up	Well-established manipulation	Yes but hyperglycosylation	Moderate and mannosylation of secreted proteins	Low cost
*Komagataella pastoris*	High cell density, easy scale-up	Well-established manipulation	Yes but hyper-mannosylation	Moderate of secreted proteins	Low cost
Filamentous fungi	Fast and high efficiency	Complex manipulation and lower transformation efficiency	Typical eukaryotic posttranslational modifications	High and efficiency secretion	Low cost
Plant cell	Safe and efficacious	Complex manipulation, long period, and lower transformation efficiency	Tailor-made glycans	High expressing	Cost and potential contamination with microorganisms
Insects	Safe for vertebrates, more demanding culture conditions	Excellent tool for recombinant glycoprotein production	Glycosylation of protein terminal with mannose glycans	High expressing but cannot be expressed continuously	High cost
Mammal cells	Slow growth and expensive nutrient requirement, limited large-scale industrial production	Complicated technology	Proper protein folding, posttranslational modifications	Moderate	High cost and potential contamination with animal viruses

The common hosts of filamentous fungi are *Aspergillus* species, *Trichoderma* species, and *Penicillium* species, such as *Aspergillus niger*, *Aspergillus oryzae*, *Aspergillus nidulans*, *Trichoderma reesei*, *Penicillium oxalicum*, and other model fungi such as *Neurospora crassa*, all of which can be used for both mechanism investigation and protein expression. Besides, *T. reesei* and *P. oxalicum* are likely to be applied in cellulases expression and plant biomass degradation, whereas *A. niger* and *A. oryzae* are often applied in food industry. What’s more, the genetic toolboxes of model filamentous fungi *A. nidulans* and *N. crassa* have been fully developed and used for the investigations of various mechanisms in filamentous fungi, especially for *N. crassa*, as the *N. crassa* Gene Knockout Library is available and brought great convenience for further studies. Filamentous fungi are not suitable for those proteins that are easily produced by other hosts with a considerable yield due to their complex and time-consuming genetic manipulations. As saprophytic fungi, most of the filamentous fungi possess the advantages of biomass degradation. Therefore, filamentous fungi are often used for the expression of CAZymes, which are responsible for the degradation of plant biomass in industrial field, thus providing clean energy by green manufacture.

**FIGURE 1:**
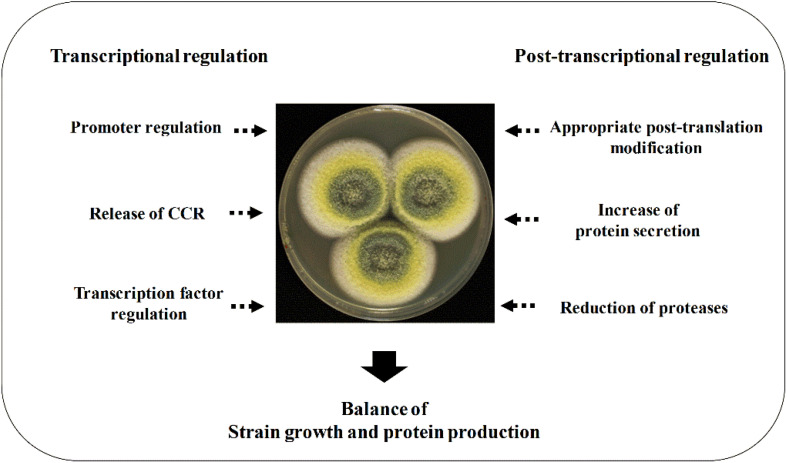
Regulation strategies for enhancing protein expression in filamentous fungi. The enhancement of protein expression at both transcriptional and translational levels is required, and a fermentation optimization is needed to balance the strain growth and protein production.

This review elucidates the regulating strategies in enzyme expression at both transcription level and protein level. Filamentous fungal cell factories produce both endogenous and heterologous enzymes. The filamentous fungi–derived CAZymes, such as cellulases, are often expressed in their native hosts. The expression of these endogenous proteins is regulated at the transcription level to a large extent under the control of carbon catabolite repression (CCR). The promoter and transcription factor regulation mechanisms as well as signal pathways of protein expression in transcription level will be highlighted in detail, which brings a better understanding of the transcription regulation and further applications in the improvement of CAZyme expression. Furthermore, the conventional regulation strategies for improving heterologous expression, such as increasing copy number, codon optimization, protein fusion expressing, and protease reduction, are also introduced briefly. The summarized regulation strategies for enhancing protein expression in filamentous fungi are shown in Figure 1.

## Transcriptional Regulation for Efficient Protein Production

Most of protein production systems for filamentous fungi cell factories require transformation methods and gene editing strategies, such as vectors, insertion manner, and selection markers, and the present studies and advances of genetic manipulations of filamentous fungi are listed in Table 2. Meanwhile, the efficiency of mRNA production and protein expression mostly depends on transcriptional regulation, which includes promoter regulation, CCR regulation, and transcription factor regulation. Herein, various regulation strategies in transcription level for protein expression in filamentous fungi are introduced.

**TABLE 2:** The advances of genetic manipulations of filamentous fungi.

Manipulations	Categories	Introduction	Host strain	References
Vectors	Autonomously replicating vector	Heterologous genes inserting outside chromosome of host cells and replicate independently in an extranuclear manner	Almost all the *Aspergillus species, Rosellinia necatrix*, *Ceriporiopsis subvermispora*	Gupta et al., 2012; Shimizu et al., 2012; Istvan et al., 2017; Li D. et al., 2017; Honda et al., 2019
	Integrated vectors	Foreign genes would be integrated into the genome and be maintained and expressed stably during mitotic and meiotic cell divisions	All the reported filamentous fungi	Istvan et al., 2017; Honda et al., 2019
Transformation methods	PEG/CaCl_2_-mediated transformation	In the presence of Ca^2+^, exogenous DNA entered into the host strain by mediation of PEG when protoplasts served as recipient cells	All the reported filamentous fungi	Jain et al., 1992; Zhang et al., 2009
	*Agrobacterium tumefaciens* mediate transformation	Exogenous genes entered into any recipient cells of the host strain such as protoplasts, mycelium, and even spores in the mediation of *A. tumefaciens*	All the reported filamentous fungi	Michielse et al., 2008; Xu and Bluhm, 2011
Selection marker	Nutrition selection	Genetic transformation selection via the remedy of exogenous substances due to the abnormal synthesis or metabolism pathway of nutrition-deficient strains, such as genes of *niaD*, *glmS*, *argB*, *amdS*, *pyrG*/*pyrF*/*pyr4*/*ura3*/*ura5*	Strain with corresponding deficiency phenotype	Navarrete et al., 2009; Liu et al., 2015; Niu et al., 2016
	Resistance selection	Selection method of strain for their growth under a certain drug concentration and show resistance when resistance gene was transferred into the host strain, such as *Bar*, *Neo*, *Hph*, *BenA*, *Ble*	Strain without corresponding resistance phenotype	Niu et al., 2016; Liu et al., 2019; He et al., 2020

### Promoter Regulation

The strategy of using known regulatory elements for protein production is widely applied in fungi cell factory, and the utilization of strong promoters in filamentous fungi can efficiently improve the transcription level of target genes. The strong promoters such as the promoters of glucoamylase gene (*glaA*) in *A. oryzae*, glyceraldehyde-3-phosphate dehydrogenase gene (*gpdA*) in *A. nidulans*, α-amylase gene (*amyB*) in *A. oryzae*, and cellobiose hydrolysis enzyme gene (*cbh1*) in *T. reesei* are most commonly used strong promoters and have been successfully applied in the efficient expression of recombinant proteins.

The original promoters of CAZymes are often inducing promoters, which called for some specific induction conditions. Thus, the strategy of converting the inducing promoter to a strong constitutive promoter is efficient sometimes and has been commonly used in the transcriptional regulation of CAZymes (Su et al., 2012). However, constitutive promoters with strong transcription abilities are not always suitable for the enhancement of proteins as recombinant proteins may be toxic for the growth of the host strain. In this case, inducible promoters would be more preferable as their controllable ability to transfer to the protein expression phase from strain growth phase (Weinhandl et al., 2014).

As a consequence, the modification of the existing promoters and new promoters mining would be better choices. The promoter series were also applied for the overexpression of the target gene (Zhang and Xia, 2016). The modification of promoters greatly enhanced the expression of proteins when the binding sites of repressors were replaced by those of activators (Zou et al., 2012; Sun et al., 2020). Native promoters with different strength were mined and used when there were no suitable or enough promoters applied in some non-model strain with industrial value (Liu et al., 2018).

### Carbon Catabolite Repression of Filamentous Fungi

As we know, the factors that affect protein expression in filamentous fungi are mainly related to the hierarchy of carbon source utilization of the strain, the signaling sensing and transduction pathways that regulate catabolites, and expression of corresponding enzymes. CCR widely exists in various microorganisms with a regulation system of carbon source utilization, which determines the utilization hierarchy of a huge variety of carbon substrates. CCR ensures the utilization of preferential carbon sources, such as glucose, and inhibits the utilization of less preferred carbon sources by repressing the expression of CAZymes required for the catabolism of a wide range of alternative carbon sources (Kiesenhofer et al., 2016). A large number of genes involved in carbon catabolism are under the control of CCR, including the industrially important CAZymes such as cellulase, amylase, and xylanase (Kunitake et al., 2019). CCR energetically selects the preferential carbon sources, helps microorganisms adapt to the environment by absorbing favorable nutrients at maximum, and reduces the cost of CAZymes synthesis when the preferred carbon source is available, which represents an economical manner for carbon catabolism regulation (Adnan et al., 2017). However, CCR is caused not only by glucose, but also by other monosaccharides. It is reported that xylose also acts as a carbon catabolite repressor, whereas the expression of enzymes for xylose utilization can also be repressed in the presence of glucose (Prathumpai et al., 2004). Besides, the expression of alcohol dehydrogenase (ADH2) of *Saccharomyces cerevisiae* was shown to be inhibited by both glucose and the acetate (Simpson-Lavy and Kupiec, 2019).

The CCR regulation system exists in various fungi and involves several regulators. CCR is mediated by the Mig1 repressor (Kayikci and Nielsen, 2015) in yeast carbon metabolism, while it is often mediated by CreA/Cre1 in most of filamentous fungi. The transcriptional repressor CreA is a C_2_H_2_ finger domain DNA-binding protein and found to mediate CCR in *A. nidulans* with a transcript of 1.8 kb in length (Dowzer and Kelly, 1989), and the orthologs were identified as CRE1 in *N. crassa* and *T. reesei* with similar functions (Strauss et al., 1995; Serna et al., 1999). CreA mediates CCR with the help of CreB–CreC deubiquitination complex, which plays a crucial role in the function and stability of CreA (Todd et al., 2000; Lockington and Kelly, 2002; Ries et al., 2016). The subcellular localization of CreA is crucial for derepression of CCR and utilization of carbon source in *A. nidulans* (Ries et al., 2016), and the detailed introduction can be found in next part. CreA functions in repressing transcription of CAZymes via directly binding to 5’-SYGGRG-3’ on the promoters of target genes or the transcription activators (Benocci et al., 2017). Besides CCR mediation, CreA also functions in hyphal growth and metabolism in *Aspergillus* species, *T. reesei*, and *Humicola insolens* (Ries et al., 2016; Xu et al., 2019).

As CreA is a repressor for CAZyme expression, strategies of CreA deletion or modification have been used for improving the expression of genes related to carbon utilization. Xylose catabolism was activated in the CreA deletion strain even under high glucose concentration, whereas the major enzymes for xylose utilization were expressed only when glucose repression was relieved in the wild-type strain of *A. nidulans* (Prathumpai et al., 2004). The significantly enhanced expression of cellulase and hemicellulase in *T. reesei* was obtained when the *cre1* gene of the mutant strain was either completely removed or partly truncated, which resulted in the derepressed cellulase expression even in the presence of glucose under both inducing and non-inducing conditions (Nakari-Setälä et al., 2009). However, persistent nuclear localization was obtained when domains of CreA in *A. nidulans* were deleted, which led to a repression of cellulase coding genes under cellulase-inducing conditions (Ries et al., 2016). More interestingly, a truncated Cre1 could turn into an activator, which functioned to activate and enhance the expression of cellulase and xylanase in *T. reesei* without causing any growth deficiencies. The truncated CreA, which served as an activator, exerted its function by locating to the nucleus and directly binding to the upstream regulatory regions of target genes under both inducing and repressing conditions, especially of the main transcription activator of the cellulases, Xyr1 (Rassinger et al., 2018).

As a consequence, the investigation of CCR is important for the regulation and enhancement of endogenous protein expression, whereas for foreign protein expression, choosing a pathway independent of CCR and a strong promoter to make the target protein expression in a constitutive manner might be a more efficient strategy for enhancing its expression level (Zhang et al., 2008).

### Signal Pathway in Filamentous Fungi

There are two prerequisites for the expression of endogenous CAZymes in filamentous fungi; one is derepression of CCR, whereas the other one is the presence of inducers. Derepression would be introduced in the following paragraphs, whereas the mechanism of how inducers function in the signaling pathway and the interactions between inducers and elements or factors of the target genes still lack of full investigation. The derepression of CCR is an essential condition for the expression of CAZymes as they can only be induced by corresponding inducers when glucose was depleted in the wild-type strain without any genetic modifications (Gancedo, 1998; Sarma et al., 2007; de Souza et al., 2013; Brown et al., 2015; Wang et al., 2017).

Derepression of CCR is achieved by depletion of favorable carbon sources or a deletion of the crucial factors related to the CCR pathway, such as CreA and other repressors that mediated CCR or PKA pathway [cyclic adenosine monophosphate (cAMP); cAMP-dependent protein kinase A (PKA)], which are responsible for the nuclear location of repressors. The repression and derepression of CCR consist of two crucial pathways, which are the AMP-activated protein kinase (AMPK) pathway and the PKA pathway (Hardie, 2010; Adnan et al., 2017; Lin and Hardie, 2017; Kunitake et al., 2019).

AMPK is regarded as a sensor and regulator of nutritious conditions of the extracellular environment as it can switch on the expression of CAZymes for alternative carbon source utilization. It is activated in the condition of low energy by sensing the cellular adenine nucleotide level, and it was first discovered in mammalian cells for its crucial role in energy sensing to regulate the energy balance of the whole body in a way of inhibiting ATP consumption and accelerating ATP production by switching on alternative catabolic pathways (Lin and Hardie, 2017). G protein–coupled receptors (GPCRs), hexokinases, and hexose transporters are all involved in glucose sensing. When high concentration of glucose was sensed by the corresponding receptors, the glucose was transported into the cell and participated in glycolysis, leading to an increased ATP level and a reduced AMP/ATP ratio, which resulted in a silenced mode of AMPK. With the consumption of glucose and ATP, the AMP/ATP ratio was increased, and the AMPK pathway was activated, which further affected the subcellular localization of CAZyme repressors directly or indirectly by exporting repressors from the nucleus, followed by the degradation in cytoplasm (Tanaka et al., 2018). Thus, the regulatory sequences of target genes were released and further bound by transcription activators, which initiated the transcription of the corresponding CAZyme coding genes (Rubenstein, 2008; Brown et al., 2013).

AMPK always exists as heterotrimeric complexes and consists of the catalytic α subunit and the β and γ regulatory subunit. The α subunit of AMPK in *S. cerevisiae*, which is encoded by s*nf1* gene with the target of Ser/Thr site, is required for the derepression of CCR (Gancedo, 1998). The repressor Mig1 is regulated by phosphorylation with the protein kinase Snf1 in *S. cerevisiae*, which is the functional homolog of CreA. Snf1 is activated at a low glucose level by glucose sensing and signaling cascades. Mig1 is phosphorylated by Snf1 and removed to cytoplasm during glucose starvation, which led to a liberation of the regulatory sequences of target genes, whereas Snf1 is inactivated by a high extracellular glucose concentration and results in nucleus localization of Mig1. And as a consequence, Mig1 binds to the regulatory element upstream of the target genes and represses their transcription (Adnan et al., 2017). Similar to yeast, the derepression of CCR in filamentous fungi is controlled dominantly by the function of MAPK. Studies show that *snfA* in *A. nidulans*, which encodes Snf1 homolog protein kinases, is required for CreA derepression and cellulase production. The absence of SnfA led to an inactivation of removing CreA away from the nucleus under the inducing condition of growth on cellulose, which indicated that the subcellular localization was an important process responding to the nutrients in the environment (Brown et al., 2013). Besides, the presence of inducers (less favorable carbon sources) and carbon starvation are also crucial for the activation of nutrient sensing kinase pathways and the release of inducer binding sites for gene induction. And CreA derepression is necessary for the induction of CAZymes (Brown et al., 2013).

The PKA pathway, which plays an antagonistic role in the regulation of CAZymes compared with AMPK pathway, is also involved in the CCR. G protein–coupled receptors, the cellular second messenger cAMP, and adenylate cyclase are all involved in the PKA pathway and affected the downstream targets by phosphorylation cascades (Nogueira et al., 2015; Yang et al., 2018). PKA is essential for CCR as its deletion leads to a misfunction of CCR with impaired nucleus location effect of repressors and partial derepression of CAZymes, which is consistent with the result obtained by the PkaA deletion strain with a varied degree of derepression of cellulase genes under different culture conditions (Kunitake et al., 2019). PKAs are involved in the regulation of various physiological processes including growth, virulence, and metabolism, and responding to the extracellular nutrient status by a series of phosphorylation cascades (Ribeiro et al., 2019). PkaA in *A. nidulans* plays a curial role in the glucose signaling pathway, and the deletion of this protein kinase resulted in an increased secretion of CAZymes but a decreased growth in the presence of inducing carbon sources (Assis et al., 2015). A mutation of amino acid site of Cre1 led to a loss of phosphorylation and the function of DNA binding, which caused a carbon catabolite derepression, and suggested phosphorylation is required for recovering the function of Cre1 from an inactive conformation (Cziferszky et al., 2002).

When a high glucose level was sensed by GPCRs or other related receptors in Ras/cAMP pathway (Wang et al., 2004), Gα subunit (catalytic subunit of G protein) was activated, and further, adenylate cyclase was activated; cAMP level was thus increased via elevated adenylate cyclase activity. The generated cAMP would bind to the regulatory subunit of PKA, which led to the liberation of PKA catalytic subunit. PKA was activated by the release of catalytic subunit and then initiated phosphorylation cascades and transmitted signals to the downstream targets, leading to the nucleus location of repressors such as CreA (Brown et al., 2014; Ziv, 2020). Phosphorylation is essential for the subcellular localization of repressors, which is crucial for repression/derepression and the transcription of the majority of CAZymes. That is, repressors such as CreA would be imported to the nucleus and repress the expression of target genes when a high extracellular glucose level was sensed, while it would also be exported from nucleus into cytoplasm and finally degraded, which led to the derepression of CCR when the energy depletion is sensed by the cell (Tanaka et al., 2018).

**FIGURE 2:**
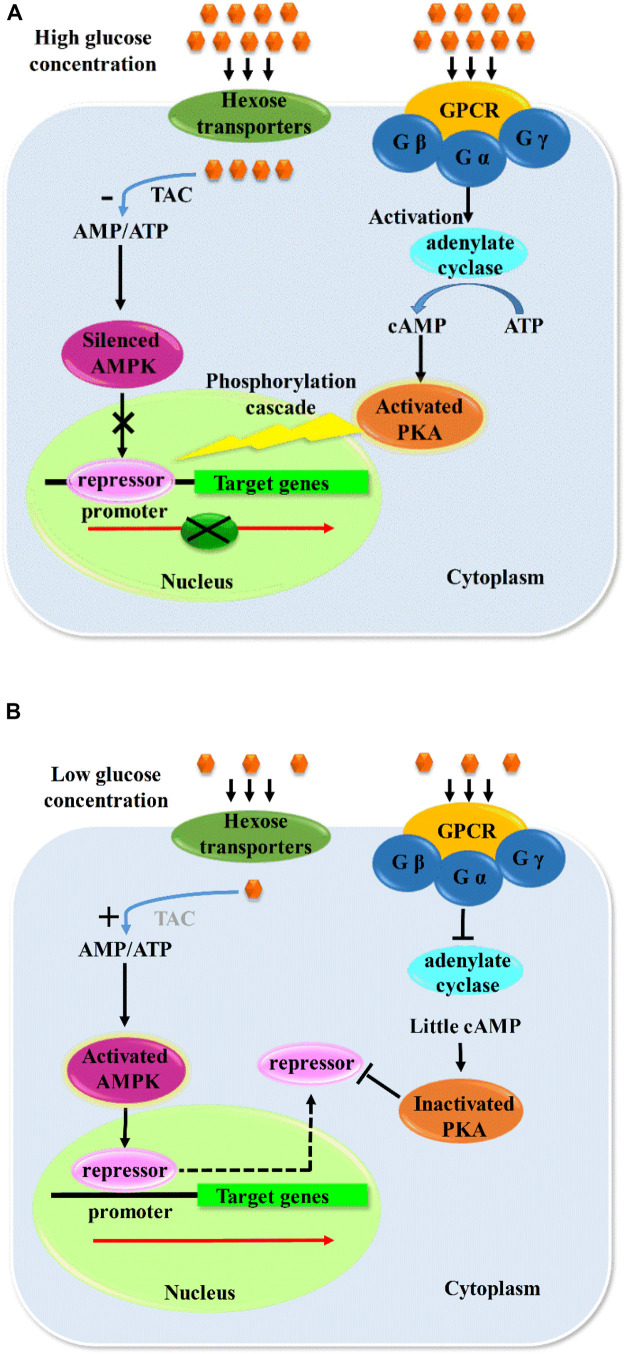
Probable model of carbon metabolism regulation. When the concentration of glucose is high outside the cell **(A)**, G protein–coupled receptor would receive and activate G protein, and catalytic subunit of which would activate adenylate cyclase and result in the increased level of cAMP, and further activate PKA and a series of phosphorylation cascade, which would cause a nucleus location of repressors. In the same time, the high concentration of glucose would accelerate the tricarboxylic acid cycle and increase the concentration of ATP, and the decreased ratio of AMP/ATP would silence the AMPK and make it unable to mediate the export of repressors to cytoplasm from nucleus, thus forming a repression model of CAZymes, and *vice versa* **(B)**. Gα, Gβ, and Gγ are the 3 subunits of G protein; Gα is the catalytic subunit of G protein. TAC, tricarboxylic acid cycle.

The receptors sense the nutrients status and transmit the signal by phosphorylation cascades to the protein kinases including AMPK and PKA pathway, followed by the direct or indirect phosphorylation effects on the specific sites of the repressors with their final subcellular localization. Some results indicated that dephosphorylation of CreA was essential for CCR in *T. reesei* (Cziferszky et al., 2002), whereas other studies declared the dephosphorylation of CreA is also found in derepressing conditions in *A. nidulans* (Alam et al., 2016). Therefore, it cannot be concluded that the role of phosphorylation is repression or derepression of CAZymes. The derepression of CCR mediated by AMPK pathway plays a positive role in the expression of CAZymes by direct or indirect phosphorylation in different hosts (Gancedo, 1998; Rubenstein, 2008; Adnan et al., 2017), but it certainly functioned via mediating the nucleus exportation of repressors. However, PKA pathway–mediated CCR has negative effects on CAZyme expression, which also regulates the catabolism via nucleus location by indirect phosphorylation of repressors as no direct interaction was found between PKA and CreA, and it was deduced that the CreA was phosphorylated via the kinases controlled by PKA, which further mediated the nucleus import of CreA (Ribeiro et al., 2019). Both PKA and AMPK pathways are indispensably involved in nutrients sensing, signal transduction, catabolism, and growth and function synergistically with the counteract effects in the regulation of CAZyme expression. The possible pathway of carbon catabolism regulation is shown in Figure 2.

### Transcription Factors Regulation

The transcription factors involved in CAZymes regulatory network are important regulatory elements in filamentous fungi. Besides CreA, which mediated CCR, there are many other transcription factors functioning as activators or repressors and playing a crucial role in the transcription regulation of CAZyme expression, most of which are Zn2Cys6-type transcription factors consisting of zinc finger DNA-binding domain and transcription activation/repression domain. The crucial activators functioning in CAZymes regulation are Xyr1 (xylanase regulator 1)/XlnR, CLR1/ClrA, and CLR2/ClrB, ClrC, and CLR-4 and also ACE2, ACE3, and AmyR, whereas the common repressors are ACE1, Rce1, and Xpp1 (xylanase promoter-binding protein 1) (Coradetti et al., 2012).

Xyr1 and its homologs are the main activators of cellulase gene expression, which play important roles in the expression of cellulolytic enzymes in many filamentous fungi, with a general consensus binding motif of the sequence GGCTRR (Castro et al., 2016; Benocci et al., 2017; Jiang et al., 2018). The overexpression of Xyr1 caused a relief from the CCR and thus produced cellulases in a constitutive expression manner, which resulted in the full expression of cellulases even on the non-inducing carbon sources in *T. reesei*, such as glucose and glycerol (Xinxing et al., 2015). AmyR is a key regulator in activating amylase expression and repressing cellulase gene expression in the meantime (Tani et al., 2001). The other activators also functioned together in the regulation of cellulases induction and positively regulated the expression of cellulases, such as CLR1/ClrA and CLR2/ClrB, ClrC, and CLR-4 and also ACE2 and ACE3 (Craig et al., 2015; Raulo et al., 2016; Liu et al., 2018; Zhang et al., 2019).

The factors such as ACE1, Rce1, and Xpp1 (xylanase promoter-binding protein 1) serve as transcriptional repressors of xylanase expression in *T. reesei* (*Hypocrea jecorina*), ACE1 can bind to the *chb1* promoter of main cellulase gene, whose deletion led to an enhancement in the cellulase and xylanase expression (Saloheimo et al., 2003; Portnoy et al., 2011). The promotion of cellulase induction and extension of induction expression process were obtained by the disruption of repressor Rce1 coding gene in *T. reesei* (Cao et al., 2017). Xpp1 could only bind to the promoters of xylanases and regulate the expression of main xylanase, without affecting the expression of cellulases (Mach-Aigner et al., 2010; Derntl et al., 2015, 2017).

Transcription regulation based on transcription factors is a useful tool in improving the CAZyme expression, such as overexpressing the transcription activators or modulating the activators into constitutive expression, and deleting the repressors if their absence does not affect the growth of strain, or downregulating when they do affect the growth. What’s more, the use of fused transcription factors to release or attenuate CCR inhibition in cellulase transcription and the modification of the existing transcription repressors to inactive mode, which lose the repression function of target genes and have no influence on the strain growth in the meantime, are promising strategies for enhancing the production of CAZymes (Alazi and Ram, 2018; Wang et al., 2019).

Artificial transcriptional factors that are fused by DNA-binding domain and effectors of different transcription factors were used in cellulase production. The strategy of fusing the ACE2 effector domain with the DNA-binding domains of CRE1 and ACE1 was used to regulate the expression of cellulase (Su et al., 2009). The binding domain of CRE1 was fused to the effector and the binding domain of XYR1, which formed a constituted expression of cellulase based on glucose serving as the sole carbon source (Zhang X. et al., 2016). Enhanced cellulase production was also obtained when a strong transcriptional activation domain was fused to the C-terminus of the natural transcription factors (XYR1, ACE2, and ACE1), followed by the transfection into hypercellulolytic strain and the replacement of natural transcription factors by homologous recombination (Zhang et al., 2018b) or by replacing natural transcription factors with minimal transcriptional activators (Zhang et al., 2018a). Randomized artificial zinc finger protein library, which was constructed via linking multiple zinc finger domain by random shuffling (Park et al., 2003), was used in cellulase expression in *T. reesei* with a significant enhancement of cellulase expression (Zhang F. et al., 2016). An elevated cellulase production was obtained in *T. reesei* when 11 amino acids of the activator ACE3 were truncated, which was probably caused by increasing the interaction with the activator XYR1 (Chen et al., 2020). The modification of ClrB with middle region removal and fusing of DNA-binding/transcriptional activation domains together led to a derepression of CCR; as a consequence, that induction of cellulase in the presence of repression carbon sources such as glucose and glycerol was obtained in *P. oxalicum* without cellulose addition (Gao et al., 2019b). The transcription factor regulation strategies are shown in Table 3.

**TABLE 3:** Strategies for transcription factors regulation.

CAZymes	Crucial factors	Regulation strategy	Host strain	Achievement	References
Cellulases	CreA, PKA	Double deletion of *creA* and *pkaA*	*A. nidulans*	Elevated cellulases but slow growth	Kunitake et al., 2019
Cellulases	Xyr1	Overexpression of *xyr1*	*T. reesei*	Full expression of cellulases on the non-inducing carbon sources	Xinxing et al., 2015
Cellulases	Cre1 and Xyr1	Overexpression of artificial activator, which fuses Cre1 binding domain to the effector and binding domain of XYR1	*T. reesei*	Constitute expression of cellulase based on glucose	Zhang X. et al., 2016
Cellulases	XYR1, ACE2, and ACE1	Fusing strong activation domain to the C-terminus of the natural transcription factors	*T. reesei*	Enhanced cellulase production	Zhang et al., 2018b
Cellulases	ACE2, Cre1and ACE1	Fusing ACE2 effector domain with the DNA-binding domains of CRE1 and ACE1	*T. reesei*	Elevated cellulases expression	Su et al., 2009
Cellulases	ACE3	Truncation of activator ACE3	*T. reesei*	Elevated cellulases production	Chen et al., 2020
Cellulases	ClrB	ClrB with middle region removal	*P. oxalicum*	Derepression of CCR and induction of cellulase under repression carbon sources	Gao et al., 2019b
Cellulase and hemicellulase	Cre1	Completely removal or partly truncation of *cre1*	*T. reesei*	Cellulases expressed under both inducing or non-inducing condition, even in the presence of glucose	Nakari-Setälä et al., 2009
Cellulases and xylanases	ACE1	Deletion of *ace1*	*T. reesei*	Increased expression of main cellulases genes and two xylanase genes	Saloheimo et al., 2003
Cellulases and xylanases	Cre1	Partially truncation of *cre1*	*T. reesei*	Cre1 turned into an activator of cellulases and xylanases by truncation	Rassinger et al., 2018
Xylanase	Xpp1	Deletion of *xpp1*	*T. reesei*	Elevated expression of xylanase and β-xylosidase	Derntl et al., 2015
Xylanase	CreA	Deletion of *creA*	*A. nidulans*	Xylanases expressed at high glucose concentration in the presence of xylose	Prathumpai et al., 2004
Amylase	AmyR	Overexpression of *amyR*	*Myceliophthora thermophila*	Increase of amylase activity by 30%	Xu et al., 2018
Lignocellulase		Deletion of *amyR*		Relief of CCR and threefold increase of lignocellulase activities	

**FIGURE 3:**
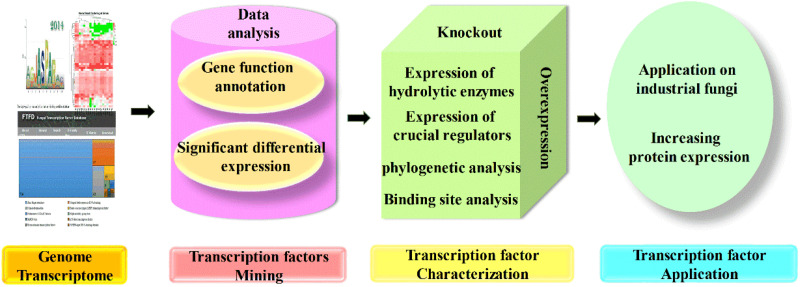
Flowchart of transcription factor mining. The mining of transcription factors often started by genome and transcriptome analysis, and candidate genes could be chosen for further characterization based on gene function and different expression level. The mutants with candidate genes knocked out; complementation and overexpression were constructed, and characterized by the expression of target regulated genes and crucial regulators, also the phylogenetic analysis and binding site analysis, which finally applied on other industrial fungi for the hyperproduction of proteins.

As mentioned previously, various transcription factors are involved in the complicated regulation network of CAZyme expression, but their mechanism investigations are still far from enough. The omics techniques play a crucial role in the investigation of transcription factors, followed by gene cloning and characterization, and their functions were finally identified by the knockout, complementation, overexpression, truncation, and even binding properties (Mäkinen et al., 2014; Li et al., 2015). Furthermore, transcriptome data are also necessary for novel regulator screening, and candidate genes were selected based on the combination of genome data and transcriptome data, which were further determined by experimental results, and finally, the underlying regulators that function in regulation of protein expression were uncovered and applied in the production of CAZymes (Derntl et al., 2017; Liao et al., 2018; Zhang et al., 2019). The flowchart of transcription factor mining is shown in Figure 3.

### Increasing Copy Number

The transcription level of exogenous genes depends more on the transcription efficiency of insertion sites in genome than copy numbers in filamentous fungi when the genome integration strategy was applied (Zoglowek et al., 2014). Unlike heterologous gene expression with high copy number plasmids in *Escherichia coli*, the selective stress is required to ensure their stable inheritance when the extrachromosomal expression manner is used in fungi (Kavanagh, 2011), which caused a less application of increasing copy number in filamentous fungi.

## Protein Level Regulation

The protein regulation includes translation and posttranslational modification, and protein secretion. The strategies during this process are mainly directed to heterologous expression, which are introduced in detail in previous studies (Sharma et al., 2009; Ward, 2011). Here are several strategies for improving protein expression in heterologous host strain.

### Increase the Translation Efficiency and Protein Secretion

Filamentous fungi can secrete protein out of the cell efficiently when endogenous genes were expressed, but the secretory ability significantly decreased when some heterologous genes were expressed. The strategy of codon optimization is crucial and useful for improvement of translation efficiency. Codon optimizing of heterologous genes according to the codon preference of host strain is an effective way and powerful tool to improve the expression efficiency of heterologous protein in filamentous fungi (Tanaka et al., 2014), which improves the translation efficiency of heterologous genes by the increase of steady-state mRNA level via the elimination of premature polyadenylation and avoidance of mRNA degradation (Li et al., 2007; Tokuoka et al., 2008). The improvement for heterologous protein focuses on the conventional strategies, which are gene fusion with a well-secreted protein and overexpression of foldases and chaperones (Archer et al., 1994). Constructing and selecting host strains with high secretory ability are also a way to improve the expression ability of foreign genes of filamentous fungi (Gombert et al., 2016). Inserting foreign genes into the downstream of the endogenous carrier protein with hyperproduction and high secretion ability by molecular manipulation, constructing the fusion expression system, and expressing the heterologous protein in a fusing manner followed by a cleavage of resulting proteins are also an effective way for improving the level of foreign gene secretion (Gustavsson et al., 2001). Human antibody fragment fused to a truncated endogenous enzyme was expressed in a fourfold protease deletion strain of *N. crassa*, which obtained a good secretion of 3 mg/L (Havlik et al., 2017). For facilitating the subsequent cleavage of the two fused proteins, a linker with proteolytic processing sites should be contained between the carrier protein and the target protein, which should be designed to allow the independent folding of the catalytic domain and the fused protein (Ward, 2011). Besides, the secretion of heterologous protein could also be increased by overexpression of foldases, signal peptides, and chaperones via facilitated protein folding (Goedegebuur et al., 2014; Nevalainen and Peterson, 2014). The application of heterologous signal peptides led to a high level of protein secretion; when enhanced green fluorescent protein served as a model, the heterologous protein human interferon β was finally successfully expressed in *Aspergillus unguis* (Madhavan et al., 2017). The porin B signal peptide in *Corynebacterium glutamicum* was used to improve the production of model protein endoxylanase with activity in high efficiency with a performance of 615 mg/L (An et al., 2013).

### Appropriate Posttranslation Modification

It is far from enough to express heterologous protein just in high transcription level and translation efficiency. The industrial and commercial values of the proteins are their structures and functional activities; therefore, appropriate posttranslation modification is crucial for the utilization of the protein produced by heterologous host strains. Unlike the simple prokaryotic expression systems, which are unable to carry out many of the posttranslation processing and lead to the secretion of inactive inclusion bodies, fungal cells have an outstanding performance of these processes. Among all the posttranslation modifications, glycosylation is very crucial for the biological activities of proteins; for example, cellobiohydrolases are typical glycoproteins, which undergo both N-linked glycosylation and O-linked glycosylation by the attachment of oligosaccharides (Stals et al., 2004; Ward, 2011). However, the overglycosylation of proteins would negatively influence enzyme activities, such as enzyme binding and protein stability. Choosing appropriate hosts for the expression of recombination protein is important because the level of glycosylation depends on host strain in a large extent. Generally speaking, the overglycosylation of filamentous fungi is less extensive than that in yeast, especially *S. cerevisiae* (Zoglowek et al., 2014). In some cases, strategies for reducing some extent of glycosylation are necessary for increasing the biology activities of proteins by site mutation, enzymatic deglycosylation, or even using glycosylation deficient strain (Kavanagh, 2011). In a word, an appropriate host strain and a reliable control system of glycosylation are both crucial during activation of heterologous protein.

### Reduction of Proteases

The occurrence of some incorrect posttranslational processing in the heterologous protein expression during the cell growth of filamentous fungi is unavoidable, such as misfolding, impairment of intracellular transport, and proteolytic degradation. Proteolytic degradation is one of the most obvious reasons for the low yields of heterologous proteins (Chung et al., 2002). Proteolytic degradation occurs not only in intracellular but also extracellular by the function of endogenous proteases produced during fungal growth (Zoglowek et al., 2014).

There are several strategies in reducing the proteolysis in host cells. Deletion of the identified protease coding genes could bring a satisfactory cellulase production compared with the parent strain (Li et al., 2019). The application of protease-deficient strains for improving the production of heterologous proteins is efficient and commonly used, although it would also bring a reduction of industrial protein production in the meantime (Wang et al., 2005). In this case, the strategy of partially inactivating some of the more prominent extracellular proteases, such as alkaline proteases or metalloproteases, which were determined in the strain, could be chosen for improving the protein production (Ward, 2011). Protease inhibitors were also used to control protease activity and reduce the proteolysis, but they could only be applied in a small-scale protein expression system (Wiebe, 2003). Besides, disruption of the coding genes of some protease regulators could also work in reducing protease activity in *Aspergillus* species. What’s more, adjusting the pH of fungal culture away from optimal pH for proteases activities and inhibiting the proteolysis could reduce the degradation of recombination protein (Braaksma and Punt, 2008).

## Challenges and Prospects for Filamentous Fungi Cell Factory

As stated above, there are so many advantages of filamentous fungal cell factory in producing industrial enzymes, and some achievements were obtained with a large amount of protein expression, which indicates filamentous fungi are a kind of efficient hosts for the production of CAZymes. The landmark studies of CAZymes are listed in Table 4. However, the mining of genome and the efforts for simplifying genetic technology are far from enough of non-model strains, such as lower transformation efficiency, multinuclear cells, lack of knowledge for available genetic engineering elements, exogenous gene insertion sites, and so on (Gupta et al., 2012; Nevalainen, 2020). It is difficult to improve the level of protein expression by a single genetic modification method. Fungal cells are always regulated by the CCR and could not express most of the CAZymes with the existence of readily available carbon sources such as glucose, whereas the lack of glucose cannot enrich the biomass of the strain, which leads to a low production of target proteins. Besides, the complex protease system of filamentous fungal cells makes them difficult to accumulate and secrete the expression products of foreign genes, resulting in low yields of target products. When some non-fungal genes from bacteria, mammals, or plants are expressed in fungal hosts, the level of protein expression would be much lower than that of fungi sourced genes (Broekhuijsen et al., 1993).

**TABLE 4:** Landmark studies of CAZymes.

CAZymes	Expression strategy	Expression level	Host strain	References
Cellulase	Truncation of functional allele of homolog catabolite repressor, Mig1	Maximum secreted protein titer were more than 14 g/L	*Penicillium funiculosum*	Randhawa et al., 2018
Cellulase	Deleting coding genes of β-glucosidase and repressor, along with overexpressing activator for blocking intracellular inducer hydrolysis and relieving the repression	Filter paper activity and extracellular protein concentration increased by up to more than 10- to 20-fold	*P. oxalicum*	Yao et al., 2015
Cellulase	Simultaneously disrupting the cellulase regulators such as repressor and protease coding genes	Extracellular secreted protein increased fivefold and lignocellulase activities significantly increased up to 13-fold,compared with the parental strain	*M. thermophila*	Liu et al., 2017
Cellulase	By the truncation of cellulase activator ACE3	Increased cellulase productivity with a maximum filter paper activity titer of 102.63 IU/mL	*T. reesei*	Chen et al., 2020
Cellulase	Deleting the serine/threonine protein kinase Stk12	Sevenfold higher of total cellulase production than that of wild type	*N. crassa*	Lin et al., 2019
Cellulase	Overexpressing endogenous β-glucosidase coding gene with two copies insertion into the chromosome of host strain	Filter paper activity of 47.0 IU/mL and pNPGase (*p*-nitrophenyl-β-glucosidase) activity of 144.0 IU/mL in fed-batch culture on lactose	*T. reesei*	Li C. et al., 2017
β-Glucosidase	Deleting CCR factor Cre1 and overexpressing a heterologous β-glucosidase coding genes	51.3-fold enhancement of β-glucosidase activity with 103.9 IU/mL	*T. reesei*	Gao, 2017
β-Glucosidase	Overexpressing β-glucosidase encoding genes	β-glucosidase activity was improved up to 65-fold with a level of 150 U/mL	*P. oxalicum*	Yao et al., 2016
Xylanase and β-glucosidase	Double deleting of the repressors CreA and CreB	Increased xylanase and β-glucosidase activities of more than 100-fold	*A. oryzae*	Ichinose et al., 2017
Thermostable xylanaseB	Heterologous expressing thermostable xylanase B in *A. niger* with endogenous strong promoter, signal sequence, and prosequence	Maximal enzymatic activity is 625 U/mL fermentation supernatant when Remazol Brilliant Blue R-D-Xylan was used as substrate	*A. niger*	Zhang et al., 2008
Amylase	Quintuple mutant modifying with the strategy of overexpressing and deleting several amylase regulators	The protein productivity and amylase activity of mutant strain were increased by 12.0- and 8.2-fold compared with wild type	*M. thermophila*	Li et al., 2020
Xylanase	Deregulating the expression of xylanase transcriptional activator XlnR and modulating the activity of the pH regulator PacC	200-fold increased xylanase activity	*A. nidulans*	Tamayo-Ramos and Orejas, 2014
α-L-Rhamnosidase		Increased α-L-rhamnosidase activity by 19-fold than that of control		
α-L-Arabinofur anosidases	Mutating alanine residue to valine of arabinose regulator AraR and overexpressing the mutant regulator	54.1-fold increase of expression	*P. oxalicum*	Gao et al., 2019a
Trehalase	Heterologous expressing high active trehalase coding gene in *A. niger* with an expression strategy of multi-copy knock-in	Titer of trehalase was up to 1698.83 U/mL	*A. niger*	Dong et al., 2019

In addition, the process of protein expression often includes two stages of strain growth and protein induction, and the strain needs to reach the maximum biomass in the growth stage by utilizing some favorable carbon sources, whereas the favorable carbon sources should be depleted, and the corresponding inducers should be added for the initiation of protein induction stage. It is obvious that monitoring the status of carbon source consumption and control of fermentation process are inconvenient. There are also some filamentous fungi that form large mycelium pellets during fermentation, which would reduce the contact between fungal cells and the surrounding medium, hindering the efficient expression and secretion of proteins (Nevalainen, 2020). The transcription regulation of CAZymes in filamentous fungi mainly focuses on the modulation of signaling pathway or modification of transcription factors, to make CAZymes express in the presence of glucose and achieve a better growth of strain and substantial biomass, thus obtaining an elevated production of CAZymes.

The strategies such as downregulation or even deletion of genes involved in PKA pathway and repressors, upregulation of AMPK pathway, and overexpressing activators are often efficient in the enhancement of CAZyme expression. However, it should be noted that PKA functions in various physical process including cell growth and metabolism, as well as the repressor CreA; their downregulation might cause deficiency in strain growth and metabolism, which must lead to a weak production of enzymes far from best. The better choice for enhancing the expression of proteins is to keep the balance between cell growth and protein expression, such as modifying a repressor into factors without negative impacts or even positive effects for the target genes or modifying the promoter regions upstream of target genes by replacing the binding sites of repressors to those of activators, and modulate the PKA and AMPK pathway to make a perfect subcellular location of repressors and incapability for repressing the gene transcription without any negative impacts on cell growth.

Moreover, the growth of the strain would change the fluidity of the medium, resulting in changes in the conditions of nutrients, oxygen, and pH; in turn, the ability of secreting foreign protein also varied with the changes of growth status and conditions (Braaksma and Punt, 2008). Therefore, the improvement with multiple tolerances of the strain by metabolic engineering strategies is essential to enhance the stability of strain in various culture environments by the relief of multiple growth stresses and so to increase the production of proteins, which needs further efforts. A further conceptual point with regard to simplifying the genetic procedures and shortening the transformation time with an acceptable transformation efficiency, multiple tolerance host strains, and the balance between cell growth and protein expression would be the main goal for the development of fungi cell factory.

## Conclusion

In summary, the present article systematically elucidated the strategies of protein expression in both transcription level and protein levels. Transcription regulations with both mechanism and signal pathway of protein expression were illustrated in detail. The strategies for producing industrial CAZymes in filamentous fungal cell factory of current studies were introduced, which are promoters and transcription factor regulation, protein expression and secretion regulation, and the balance of strain growth and protein expression. Although the catabolism regulatory network in filamentous fungal cells is really complex, our understanding of signaling pathways and mechanisms for CAZymes induction and also strategies of protein expression regulation improved the cognition of protein expression in filamentous fungi, which would benefit the investigations of CAZymes or filamentous fungi protein expression system.

## Author Contributions

TZ was responsible for the literature survey and the writing of whole manuscript. HL was responsible for the revise of manuscript. BL and CL were responsible for supervision. All authors contributed to the article and approved the submitted version.

## Conflict of Interest

The authors declare that the research was conducted in the absence of any commercial or financial relationships that could be construed as a potential conflict of interest.
